# Phosphoproteomic dysregulation drives tumor proliferation in Cushing’s disease

**DOI:** 10.1101/2025.09.28.679056

**Published:** 2025-09-30

**Authors:** David T. Asuzu, Dustin Mullaney, Debjani Mandal, Diana Nwokoye, Sheelu Varghese, Daniela Tortoza Lopez, Dhruval Bhatt, Kory Johnson, Abdel Elkahloun, Zied Abdullaev, Kenneth Aldape, Dragan Maric, Clarisse Quignon, Nasir S. Malik, Joseph P. Steiner, Yan Li, Susan Wray, Lynnette K Nieman, Christina Tatsi, Prashant Chittiboina

## Abstract

Pituitary adenomas constitute up to 20% of primary brain tumors, yet somatic mutations are only found in 15% of pituitary adenomas. Epigenomic dysregulation has been proposed as a tumorigenic mechanism in pituitary adenomas causing Cushing’s disease (CD). We created paired datasets of human CD adenomas and en-route margin adult human pituitary glands and assayed their chromatin accessibility, DNA methylation, transcriptomic, proteomic and phospho-proteomic landscapes. In CD adenomas, we found epigenetic reactivation of a neurodevelopmental phosphoprotein program typically lost in the post-natal pituitary gland. CD cells overexpressed *PPP1R17* , a potent endogenous inhibitor of the ubiquitous protein phosphatase PP2A. Mechanistically, *PPP1R17* overexpression in normal murine pituitary cells recapitulated the adenoma phenotype, and PPP1R17-mediated tumorigenesis was reversible using an FDA-approved small molecule PP2A agonist both in-vitro and in-vivo. Our findings highlight aberrant peptide phosphorylation as a targetable mechanism in CD.

## Full Text Availability

The license terms selected by the author(s) for this preprint version do not permit archiving in PMC. The full text is available from the preprint server.

